# Discrimination of object information by bat echolocation deciphered from acoustic simulations

**DOI:** 10.1098/rsos.231415

**Published:** 2024-01-24

**Authors:** Yu Teshima, Mayuko Mogi, Hare Nishida, Takao Tsuchiya, Kohta I. Kobayasi, Shizuko Hiryu

**Affiliations:** ^1^ Acoustic Navigation Research Center, Doshisha University, Kyoto 610-0321, Japan; ^2^ Faculty of Life and Medical Sciences, Doshisha University, Kyoto 610-0321, Japan; ^3^ Faculty of Sciences and Engineering, Doshisha University, Kyoto 610-0321, Japan; ^4^ Project Team for System Development of Marine Environmental Impact Assessment / SIP Ocean Program, Japan Agency for Marine-Earth Science and Technology (JAMSTEC), Yokosuka 237-0061, Japan

**Keywords:** occlusion spots, FDTD simulation, target discriminations

## Abstract

High-precision visual sensing has been achieved by combining cameras with deep learning. However, an unresolved challenge involves identifying information that remains elusive for optical sensors, such as occlusion spots hidden behind objects. Compared to light, sound waves have longer wavelengths and can, therefore, collect information on occlusion spots. In this study, we investigated whether bats could perform advanced sound sensing using echolocation to acquire a target's occlusion information. We conducted a two-alternative forced choice test on *Pipistrellus abramus* with five different targets, including targets with high visual similarity from the front, but different backend geometries, i.e. occlusion spots or textures. Subsequently, the echo impulse responses produced by these targets, which were difficult to obtain with real measurements, were computed using three-dimensional acoustic simulations to provide a detailed analysis consisting of the acoustic cues that the bats obtained through echolocation. Our findings demonstrated that bats could effectively discern differences in target occlusion spot structure and texture through echolocation. Furthermore, the discrimination performance was related to the differences in the logarithmic spectral distortion of the occlusion-related components in the simulated echo impulse responses. This suggested that the bats obtained occlusion information through echolocation, highlighting the advantages of utilizing broadband ultrasound for sensing.

## 1. Introduction

Humans rely heavily on visual information to understand their surroundings. As a result, various technologies have been implemented to better comprehend the surrounding environment, with a particular emphasis on visual sensors and visualization technology. Recent advances in computer vision using deep learning combined with multi-modal sensors have allowed optical cameras to serve as primary external sensors for object detection [1,2]. Despite efforts to detect three-dimensional information from two-dimensional images obtained by visual sensors, similar to real-world objects [3–5], one of the main challenges faced by vision sensors involves overcoming the misrecognition of transparent objects, such as glass or obtaining information on occlusion spots caused by blocked light. In particular, acquiring information about occlusion spots, such as the back of an object or behind an object, remains a significant challenge in designing sensor systems.

Due to its high attenuation, sound (ultrasonic) sensing has the disadvantage of a shorter propagation distance in air compared to light. One of the physical properties of sound wave propagation is the phenomenon of sound diffraction, where sound travels around the back of an object and propagates into its interior, which is not observable externally or visually. This characteristic is specific to acoustic sensing and is not found in light or lasers due to their shorter wavelengths. Therefore, by utilizing this diffraction phenomenon of sound waves, an acoustic technique for acquiring information on the back of an object (occlusion information) may be obtained [6,7].

Echolocating bats use active sound-based sensing (echolocation) by emitting ultrasound and utilizing the echoes from their surroundings. For example, bats use the time difference between the emitted pulse and returning echo (echo delay) to derive the target distance [8]. As a neural basis for distance measurement, delay-tuned neurons, which respond specifically to two sound stimuli with a specific time difference, are located in the ascending auditory pathway above the inferior colliculus. Certain bat species have topological substructures, or target range maps, in which delay-tuned neurons are uniformly aligned in the auditory cortex [9–11]. However, behavioural experiments have shown that bats using wideband frequency-modulated (FM) ultrasound for echolocation can obtain not only the distance information from the echo delay to the target [8], but also complex features regarding the target such as the texture, size, and three-dimensional shape [12–17]. *Eptesicus fuscus* can discriminate a smooth sphere object from a textured object by echolocation while free-flying, and studies have suggested that the difference in the spectra contained in the broadband echoes may be used as a cue [14]. Thus, the results of previous studies on the echolocation behaviour of bats suggested that sound-based sensing may provide more comprehensive information than what has been commonly assumed.

In this study, we assessed whether broadband ultrasound sensing can capture the occlusion information of targets, which is challenging to detect with visual sensors using linear light. Specifically, our investigation focused on determining whether bats use this information through echolocation, which has not been addressed before in bat behavioural experiments. To examine the ability of bats to gather target occlusion information through echolocation, tests were conducted on *Pipistrellus abramus*, which use FM ultrasound for echolocation, to distinguish between targets with high visual similarity from the front, but with different backend geometries, i.e. occlusion spots and textures. Although bats were observed to utilize the features of spectral patterns in echoes as cues [12–17], analysing the actual echoes acquired by the bats was challenging owing to the limitations of various measurement systems. This aspect has not been investigated extensively. Therefore, the echoes produced by these targets were calculated precisely using three-dimensional acoustic wave equation finite difference time domain (WE-FDTD) simulations [18–21]. The proposed approach in this study combined behaviour and simulation, allowing for a detailed analysis of the acoustic cues that bats use to obtain information through echolocation, highlighting the advantages of using broadband ultrasounds for sensing.

## 2. Material and methods

### 2.1. Animals

In the behavioural experiment, seven female adult *Pipistrellus abramus* bats were used, which were captured in Kyotanabe City, Kyoto Prefecture, Japan, on May 14, 2021. This species of bat vocalizes FM pulses for echolocation and is suitable for training two-alternative forced choice while moving toward a target without flying. The bats were kept in a rearing cage and provided with free access to food (mealworms) and water. The training of each bat started on different dates, with the first bat starting training on 19 May 2021, and the last bat completing the test on 22 October 2021. Throughout the training period, the bats were provided drinking water in the rearing cage, but food was mainly provided as a training reward. To ensure their well-being, the body weights of the bats were measured daily, and the number of rewards was carefully adjusted to maintain their weight.

All experiments involving animals were conducted following the Ethics Review Committee of Doshisha University, and the regulations were pre-approved (No. A22015) by the Animal Experiment Committee of Doshisha University.

### 2.2. Experimental setup and procedures

#### 2.2.1. Behavioural experiment

Training and testing were performed in a soundproof box using infrared light (380 × 500 × 600 m; L × W × H), which was fully isolated from light, to prevent the bats from visually observing the targets. Inside the box, a video camera (The Imaging Source DMK 23UP1300, The Imaging Source Asia Co., Ltd., Taipei, Taiwan) and a microphone (Avisoft-Bioacoustic CM16/CMPA, Avisoft Bioacoustics e.k., Glienicke/Nordbahn, Germany) were installed on the ceiling to record the behaviour of the bats and the ultrasound emissions, respectively. The video camera recorded at a frame rate of 30 fps, and the data were analysed using motion capture software (Ditect Corporation, Dipp-Motion version 1.1.31, Tokyo, Japan) to reconstruct the movement trajectories of the bats. For acoustic simulation, the direction of the echolocation pulses emitted by the bats was substituted with the directions of the bat head positions, which was calculated from the obtained video data [22]. The directions were represented as a straight line passing through the tips of the bats' mouths and the centers of their heads. The audio data, which captured the ultrasound emissions, were sampled at 192 kHz.

#### 2.2.2. Targets for identification

**Figure 1.**
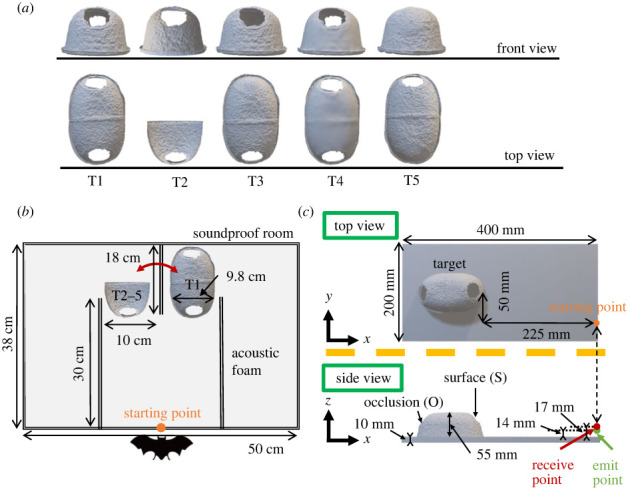
(*a*) Front and top views of the five targets used in the experiment: T1 operant stimulus target; (*b*) top view of the experimental system of the two-alternative forced choice test; (*c*) top and side views of the digital three-dimensional target (T1) used to calculate echo simulation in the simulation space.

Five types of polylactic acid resin targets were prepared in this study. To efficiently explore the ability of the bats to discriminate between targets using their natural behaviour whenever possible, we used operant stimuli targets consisting of domes with two holes for entry and exit in the front and back, mimicking the shape of paper domes that bats typically use as a roost in their rearing cage. A paper dome (146 × 98 × 54 mm) was digitally modelled using a three-dimensional scanner (EinScan SE, SHINING 3D Tech Co., Ltd., Hangzhou, China) at a resolution of 1.3 megapixels. The three-dimensional digital models were then edited using Netfabb (Autodesk Inc., California, USA) and printed with polylactic acid resin using a three-dimensional printer (Replicator +, MakerBot Industries, LLC, New York City, USA) with a spatial resolution of less than 0.1 mm. Five types of dome-shaped targets were created (figure 1*a*). The first target (Target 1, T1) was the original dome-shaped target with two holes, with a surface roughness (Rz) of 3.3 mm, and the bats were trained to go to this target. Target 2 (T2) replicated the original surface roughness of T1, but with the back half removed, which served as the target's occlusion spot, to investigate if the bats could distinguish it from T1. Target 3 (T3) also retained the original surface roughness of T1, but the hole of the occlusion spot was plugged. To explore how texture information affected the echo component, Target 4 (T4) was created, which maintained the shape of T1 but had a smoother surface (Rz = 0.0 mm). Finally, Target 5 (T5) had the same surface roughness as T1, but the front hole was plugged. Therefore, T2 and T3 had a similar texture, but differed in their structures of the occlusion spot, while T4 mirrored the structure of T1. As a result, all targets were designed with a high degree of similarity to T1 when viewed from the front.

#### 2.2.3. Training

The training procedure for guiding the bats toward target T1 consisted of two phases. During the first training phase, the bats were randomly placed on the floor inside a soundproof box. Upon walking forward T1, the bats were rewarded with mealworms. If the bats climbed up the wall of the soundproof box or failed to move toward the target after 5 min, the trial was stopped and the bat was returned to the starting position by the experimenter. The number of trials per day was adjusted to maintain the bat's weight according to weight loss on that day, and each bat underwent training sessions lasting between 30 min to 1 h daily. If the bats did not continuously move from the starting position, the training session was terminated, and the bats were provided mealworms to restore their weight to the previous day's level. Two bats were declared operant failures, as they did not exhibit any inclination of moving toward target T1 after 6–10 days in the first training phase. Therefore, the second training phase proceeded for the remaining five bats once the cumulative success rate of the first training phase exceeded 75% for three consecutive days.

The second training phase was conducted in the same soundproof box with a wall constructed from sound-absorbing material. This wall was placed in the middle of the path, dividing the path into two sections: left and right portions (figure 1*b*). Note that the aisle had a width of 10 cm, and that the largest width of the target was 9.8 cm. This resulted in free space of 1 mm on either side of the target. In addition, because the target was domed, space existed for sound waves to propagate between the walls of the aisle and sides of the target, in addition to the space above the target. In the box, long-wavelength light with filters (to remove wavelengths below 650 nm) was used to minimize the visual target recognition by bats [23]. Target T1 was placed on either side of the wall following a pseudo-random sequence, and the bats were positioned at the starting point and fed mealworms if they approached the target. To prevent habituation, two T1 samples were fabricated using a three-dimensional printer and used alternately. After each trial, the targets were disinfected with alcohol to eliminate odour cues.

#### 2.2.4. Test

Individuals with a cumulative success rate exceeding 75% in the second phase of training (electronic supplementary material, figure S1) were subjected to the test trial, which was conducted using a two-alternative forced choice paradigm. The bats were not rewarded in the test trials. Therefore, to ensure their motivation toward the target, the second stage of training was continued with test trials intermixed with the training trials. This pattern consisted of two training trials, followed by a repeated test trial, and this change in reward did not affect the behaviour of the bats. In the test trials, the T1 (operant stimulus) was placed on either side of the wall using a pseudo-random sequence, and on the other side, a target ranging from T2 to T5 was placed. If the bat approached T1, it was considered the correct choice. The number of test trials conducted each day varied according to the daily weight and motivation of the bats, but generally ranged between 5 and 10 test trials per day. A total of 15 test trials were performed for each of the four test conditions (four combinations consisting of T1 versus T2, T1 versus T3, T1 versus T4 and T1 versus T5) for each individual bat. A total of four test conditions were conducted, however, only one condition was carried out on all five bats, with the remaining three conducted on only three bats. The reason for this was that two of the five bats were not motivated to move toward the targets and remained in the starting position from the training trial as well as during the test trial, negatively affecting their test performance.

#### 2.2.5. Echo simulation

Echoes from each target that returned to the positions of the bats during the test trials were simulated using the wave equation finite difference time domain (WE-FDTD) method, in which the wave equation was directly discretized [18–20]. Unlike the standard FDTD method [24,25], the WE-FDTD method did not require the calculation of particle velocities and permitted the simulation of sound wave propagation with lower PC memory requirements compared to the standard FDTD method. The FDTD method also enabled the simulation of impulse sound wave propagation, which was challenging in the measurement experiments, and allowed for calculations of the echo impulse response of the target.

For this study, a simulation space of 420 × 220 × 80 mm was established (figure 1*c*) with a perfectly matched layer boundary condition [19,26]. The simulation space incorporated a floor thickness of 10 mm with a reflection coefficient of 0.99, where the dome target was positioned. The three-dimensional digital models of the targets employed for three-dimensional printing in the behavioural experiment were voxelized using SUF2BOX [27] and introduced into the simulation space (figure 1*c*). The targets were meshed with a cubic of 0.2 mm per side. The simulation codes were the same as those validated in a previous study [21]. In the simulation space, the speed of sound was set to 340 m/s, the spatial resolution was 0.2 mm, the Courant Friedrichs Lewy number was 0.5, and the calculated time was 2.65 ms. An impulse waveform was emitted from a source position 14 mm above the floor, corresponding to the position of the mouth of *P. abramus* (electronic supplementary material, figure S2). This mirrored the bat's ultrasound pulse emission from the starting point as illustrated in figure 1*b*. The receiving point was also set at the same location, but 17 mm above the floor (figure 1*c*). It should be noted that in this simulation, the effect of the bat's head transfer function was not taken into account. Therefore, the echoes reaching the left and right ears were not calculated separately, but rather the simulation was performed with a single point of reception. The echoes measured in the floor-only space without targets were calculated in advance to ensure that the impulse response from the target on the floor was calculated only by subtracting the echoes in the floor-only space from those obtained when the three-dimensional digital models of the targets were placed on the floor. This approach was adopted because the sound sources used in this simulation were omnidirectional. Therefore, unlike when bats emit directional pulses towards a target, sound was also emitted directly downwards from the source. As a result, the simulation echoes received at the receive point were observed to contain, first and foremost, the largest echoes directly reflected from the floor. It was necessary to remove this to ensure the capture of echoes from targets on the floor, which is the information the bats obtained from the echolocation. This allowed for visualization and analysis of the differences in echo components due to targets with high spatio-temporal resolution, and was particularly suitable for the impulse response of the echoes using an impulse waveform as a source. Furthermore, by convolving the impulse response with an arbitrary signal, actual observed waveforms were simulated. In this study, in addition to the impulse response, the frequency-modulated (FM) echoes from the target were calculated by convolving FM sounds using equation (2.1), similar to that of the FM pulse of echolocating bats [28]

2.1 $$\begin{array}{c} \;f(t)=\frac{f_{0}}{f_{0}-f_{1}}\left\{(f_{0}-f_{1}){\left(\frac{af_{1}}{f_{0}}\right)}^{t}+(1-a)f_{1}\right\}, \end{array}$$

where *a* = 0.005, *f*_0_ = 40, and *f*_1_ = 80 kHz, corresponding to *Pipistrellus abramus*. The pulse duration was set to 10 ms.

Previous research has suggested that bats rely on spectral patterns to distinguish variations in texture and shape [12,14,15]. Therefore, we employed the time-varying differences in spectral patterns (i.e. spectrogram) to calculate the changes in echoes from each simulated target. The differences in echoes were calculated according to the following logarithmic spectral distortion (LSD) in equation (2.2):

2.2 $$\begin{array}{c} \mathrm{LSD}=\underset{n,l}{\sum }20log\frac{\left|X_{n,l}\right|}{\left|{\hat{X}}_{n,l}\right|}, \end{array}$$

where *n* is the time frame, *l* is the frequency bin, and $|X_{n,l}|$ and $|{\hat{X}}_{n,l}|$ denote the amplitude spectrum. The window length for the spectrogram was 1024 and the shift was 24. In this study, the difference between the echoes from targets T1 and T2–T5 was quantified by assigning the amplitude spectrum of the echoes from T1 to $|{\hat{X}}_{n,l}|$, and the amplitude spectrum of the echoes from T2 to T5 was assigned to $|X_{n,l}|$.

## 3. Results

### 3.1. Two-alternative forced choice test

**Figure 2.**
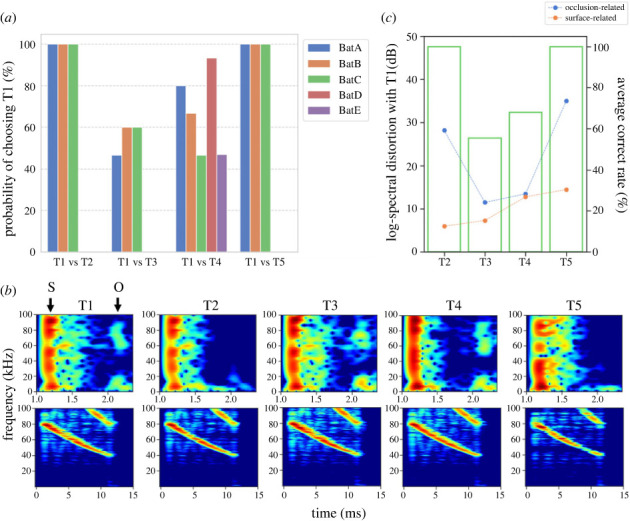
(*a*) Probability of choosing T1 (operant stimulus) for each bat in the four different two-alternative forced-choice tests; (*b*) spectrogram of the simulated echo impulse response (top panel) and FM convolved echo (bottom panel) for each target (T1–T5) (S: surface-related echoes, O: occulusion-related echoes); (*c*) average success rate (choosing T1) for each test trial (bar graph), surface-related, and occlusion-related log-spectral distortion for each target (T2–T5 and T1; line graph).

Five female *Pipistrellus abramus*, designated A, B, C, D and E, were successfully trained to move toward target T1 in the second phase of training, however, two of the seven bats that did not move toward the target dropped out during training. Five trained bats underwent 15 test trials for T1 versus T4, and three bats (A, B, and C) were subjected to T1 versus T2, T3, and T5 trials, respectively. The results showed that all individuals identified targets T2 and T5 from T1, with an average accuracy of 100%, but had difficulty identifying T3 (average 55.7%) (figure 2*a*). The higher discrimination accuracy of target T2 with the back half removed indicated that the bats could detect the occlusion spot information through echolocation. T3, with a blocked occlusion spot, was difficult for the bats to identify. Target T4, which had the same shape as T1 but with a different texture, presented an average correct response rate of 68%, but with large individual differences: two individuals exhibited high rates of 93% and 80%, indicating their ability to discriminate between the targets (electronic supplementary material, figure S3).

### 3.2. Echo analysis by acoustic simulation

The differences in bat discrimination performance among the targets were expected to be associated with differences in the echoes originating from the operant stimulus, T1. Therefore, we simulated the echo impulse responses from each target reaching the bats and examined the differences between the targets based on the spectrograms (figure 1*c*). Assuming that the bats emitted a pulse and received an echo at the starting point, the simulated impulse responses of the echoes for the five targets were obtained, as shown in the top panels in figure 2*b*. In addition, the FM sound echoes, which were produced by convolving the echo impulse responses with the sounds simulating the typical FM pulse of *Pipistrellus abramus*, are shown in the bottom panels of figure 2*b*. Although not apparent from the spectrogram of the latter FM sound echoes, the spectrogram of the former echo impulse response showed a clear difference in the echoes of the respective targets. Namely, the first echo component was observed with an echo delay of approximately 1.2 ms. This approximately corresponded to the distance between the surface of the target and receiving point, thereby indicating the surface-related echo. This echo component was defined as surface-related echoes. This was followed by a second prominent echo component that emerged with an echo delay of approximately 2.2 ms. It was observed prominently at T1, T3, and T4. The second prominent echo component was considered to encompass echoes entering through the front hole and reflecting off the interior rear surface of the target. This also included target information regarding the back side of the structure, specifically the occlusion spot. This echo component was defined as occlusion-related echoes in this study.

We then evaluated the differences in echo components between each target and T1 with respect to the first echo component associated with the surface, and the occlusion spot second echo component associated with the target. This evaluation employed LSD (figure 2*c*). We found that the surface-related components were uniformly larger in LSD for T2, T3, T4, and T5 (T2 = 5.98 dB, T3 = 7.32 dB, T4 = 12.8 dB and T5 = 14.5 dB, orange plots in figure 2*c*), whereas the occlusion-related components exhibited lower LSD values for T3 and T4 (T3 = 11.5 dB and T4 = 13.5 dB) compared to T2 and T5 (T2 = 28.2 dB and T5 = 35.0 dB) (blue plots in figure 2*c*). The differences in LSD were predominantly influenced by the occlusion-related component rather than the surface-related component. Furthermore, the relationship between the bat's correct response percentage in the two-alternative forced choice test trials for each target aligned with the echo LSD for the occlusion-related component (figure 2*c*). Hence, a lower LSD value for the occlusion-related component corresponded to a lower correct response rate of the bats in the discrimination test.

## 4. Discussion

### 4.1. Utilizing information obtained from occlusion spots in bats

We found that the discrimination performance of the bats was related to the differences in echo impulse responses, revealing their ability to distinguish between variations in target occlusion spot structures by echolocation. These findings suggested that bats leveraged sound sensing to obtain information that proved challenging to acquire visually.

Traditional light-based sensing, such as cameras, face limitations in obtaining occlusion-related information due to the shorter wavelength of light compared to sound, preventing it from going behind an object. With the increasing need to improve the safety of autonomous vehicles and robots, recent engineering efforts have sought to overcome this limitation by acquiring occlusion information using cameras and lasers [4,29–32]. Sound waves have longer wavelengths than light and can penetrate objects and reach behind them. However, it has remained unclear whether bats utilize these properties during echolocation. In this study, we showed that the differences in echo components, LSD, including the occlusion spot information, were associated with the bats' discrimination performance, indicating that bats used this information as a cue.

High echo-delay resolution is necessary to perceive target shape and texture, and previous behavioural experiments have reported an echo delay accuracy of 2 μs in big brown bats using FM sound [33]. In these extremely short delay separations, the notch in the spectrum created by the superposition of the two FM sounds was considered a cue for bats [12,33]. In this study, the impulse response of the echoes received by the bats clearly showed that they received echoes from the target surface, followed approximately 1 ms later by echoes containing occlusion spot information. The echo components at approximately 1 ms intervals observed in the impulse response were sufficiently discriminative compared to the echo delay resolution reported for FM bats [12], suggesting they could serve as a cue for *P. abramus*.

Notably, the impulse response of the surface-related echo components exhibited notches around 60 kHz, except for T4, which maintained the shape of T1 but featured a smoother surface. Therefore, the notches were presumed to be related to the surface texture of the target [34]. Interestingly, the results showed individual differences in correctly identifying T1 versus T4 (electronic supplementary material, figure S3), and bats A and D achieved high percentages of correct responses (93% and 80%), while bats C and E performed at a chance level. These results suggested that the cues used to identify T1 differed among the individuals. Hence, the two individuals with the highest correct response rate used the notches with the surface-related echo component as a cue to discriminate between T1 and T4. Therefore, if the occlusion-related echo component was used as a cue to identify T1, detecting differences between the echoes of T1 and T4 would be challenging.

Furthermore, none of the bats managed to discriminate T3, which contained a blocked hole in the occlusion spot, from T1. Figure 2*b* shows that the impulse response of T3 contained a clear occlusion-related component, similar to T1, and a notch around 60 kHz in the surface-related component. As a result, the difference between the presence and absence of the hole at the back could not be clearly confirmed by the impulse response within this time frame. This indicated that echolocation did not always yield information from the occlusion spots, and depended on the amount of information contained in the echo. Therefore, acoustic differences may not necessarily correlate with visually determined differences, as exemplified by bats colliding with mirrors [35].

Conventional acoustic measurement methods, which involve placing targets in a physical space and measuring the echoes, have difficulty analysing the impulse responses. By introducing the experimental space into the acoustic simulation space, the cues used by bats could be examined in more detail, which may help to elucidate the information acquired by echolocation.

### 4.2. Accumulation of information by sensing while moving

The abovementioned analysis primarily focused on the echo information acquired from the bats’ echolocations at the starting point. However, the bats accumulated information and gained confidence in their ability to sense differences from T1, while walking and repeatedly receiving information regarding their targets through echolocation. Therefore, we used actual moving trajectories and pulse emission timing of the bats to examine how echo information changed as the bat walked toward the target in terms of LSD.

**Figure 3.**
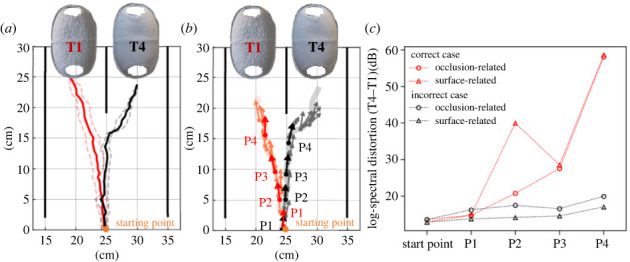
(*a*) Trajectory of the bats toward targets T1 (red) and T4 (black) in the T1 versus T4 test trial, where the solid and dotted lines represent the mean and standard deviation, respectively. Because T1 and T4 were randomly positioned on the left and right sides of the experimental setup, the trajectories for the trials in which T1 was positioned to the right were inverted. (*b*) The pulse position and direction of the bats during one trial toward T1 and one toward T4 in the T1 versus T4 test trial. (*c*) Log-spectral distortion (T4–T1) of the echoes calculated by acoustic simulation at positions P1–P4 (correct shown in red and incorrect shown in black), shown in (*b*).

Figure 3*a* presents the outcomes for the top two individuals (bats A and D) with the highest percentage of correct responses in identifying targets with different surface textures (T1 versus T4, electronic supplementary material, figure S3). Notably, when T1 was placed in the right corridor, the trajectory direction was reversed from left to right, ensuring that T1 was placed in the left corridor during all trials. The locations and directions of the pulse emissions produced by the bats (head direction) for this point in time are shown in figure 3*b* for both typical correct and incorrect trials. In addition, figure 3*c* presents the changes in LSD between T1 and T4, which were obtained by selecting four representative pulse emissions for each correct and incorrect case (solid arrows in figure 3*b*). The spectrograms of the impulse responses for the four echoes are shown in electronic supplementary material, figure S4. In the correct case, the LSD value drastically increased when the bat moved in a straight line toward T1. By contrast, the trajectories of individuals who were unsure of their decision moved linearly in the centre, and the LDS did not change significantly in this case. This suggested that the decision making of bats regarding their movement trajectory was related to changes in LSD as they moved. The changes in the impulse response of the echoes, obtained by combining behaviour and simulation, were also useful for studying the spontaneous decision process of the bats.

## 5. Conclusion

In this study, we examined the interesting phenomenon of bats using information obtained from visual occlusion spots and texture for object identification via echolocation. The results revealed that bats were able to discriminate differences in target occlusion spot structure and texture through echolocation. Furthermore, the discrimination performance of bats was related to differences in the logarithmic spectral distortion of the occlusion-related components in the simulated echo impulse response.

Despite the importance of analysing the echoes received by bats to understand their acoustic sensing tactics, certain technical limitations related to acoustic measurements have remained. Recently, we developed a new acoustic simulation method that is able to obtain echoes received by bats with high accuracy [36]. In the present work, we introduced this experimental system into the digital space and calculated echoes with the constructed acoustic simulation, which were difficult to obtain with real measurements, allowing us to investigate the acoustic cues that bats obtain through echolocation. Furthermore, this study used acoustic simulations to analyze how target information was updated by echolocation behaviour, providing additional insights into bat echolocation tactics. By learning from bat echolocation and utilizing the advantages of broadband ultrasonic wave propagation, we could discover new applications for ultrasonic sensing, such as sensing occlusion spots.

## Data Availability

Data are available at figshare (https://doi.org/10.6084/m9.figshare.21834969) [37], and data and relevant code for this research work are stored in GitHub: https://github.com/tsmyu/Effect-of-bat-pinna-on-sensing-using-acoustic-finite-difference-time-domain-simulation and have been archived within the Zenodo repository: http://dx.doi.org/10.5281/zenodo.10405556 [21]. Supplementary material is available online [38].
